# Non-invasive qualitative and semiquantitative presurgical investigation of the feeding vasculature to intracranial meningiomas using superselective arterial spin labeling

**DOI:** 10.1371/journal.pone.0215145

**Published:** 2019-04-09

**Authors:** Ulf Jensen-Kondering, Michael Helle, Thomas Lindner, Olav Jansen, Arya Nabavi

**Affiliations:** 1 Department of Radiology and Neuroradiology, University Hospital Schleswig-Holstein, Campus Kiel, Germany; 2 Philips GmbH, Innovative Technologies, Research Laboratories, Hamburg, Germany; 3 Department of Neurosurgery, Klinikum Nordstadt, Hannover, Germany; George Washington University, UNITED STATES

## Abstract

**Background:**

Intracranial meningiomas may be amenable to presurgical embolization to reduce bleeding complications. Detailed information usually obtained by digital subtraction angiography (DSA) on the contribution of blood supply from internal and external carotid artery branches is required to prevent non-target embolization and is helpful for pre-surgical planning.

**Purpose:**

To investigate the contribution of the feeding vasculature to intracranial meningiomas with superselective arterial spin labelling (sASL) as an alternative to DSA.

**Material and methods:**

Consecutive patients presenting for meningioma resection were prospectively included. sASL perfusion images acquired on a clinical 3T MRI scanner were independently rated by two readers. Contribution of the external carotid artery (ECA), internal carotid artery (ICA) and vertebral/basilar artery (VA/BA) was rated as none, <50% or >50%. Correlation of sASL was performed in two patients undergoing DSA.

**Results:**

32 patients (61 ± 13 years) harboring 42 meningiomas could be included. sASL was technically successful in all patients. 19 meningiomas had ICA dominant supply, 19 had ECA dominant supply. One meningioma had mixed supply and in three meningiomas a perfusion signal could not be detected. While exclusive unilateral ECA supply was common (n = 14) and exclusive unilateral ICA was rare (n = 4), mixed supply from multiple vessels (n = 20) was a frequent finding. Interrater agreement was substantial (κ = 0.73). Agreement with DSA was perfect within our predefined categories.

**Conclusion:**

sASL is able to identify the presence and extent of the feeding vasculature in intracranial meningiomas.

## Introduction

Meningiomas are the most common benign tumors of the central nervous system. Although malignant meningiomas and malignant transformation are rare, space occupying or symptomatic meningiomas may require resection [1]. Meningiomas are richly vascularized lesions and thus are amenable to presurgical embolization to reduce bleeding complications [2]. Detailed information on the contribution of blood supply from internal and external carotid artery branches are helpful for pre-surgical planning and required to prevent non-target embolization [3]. This is usually obtained by digital subtraction angiography (DSA). However, DSA utilizes ionizing radiation and iodinized contrast agent. Further, DSA is an invasive procedure and bears the risk of stroke, hemorrhage and vessel perforation [4].

CT or MRI based angiography techniques like CTA or TOF are non-selective and cannot quantitatively differentiate the contribution of multiple feeding vessels [5,6]. Perfusion techniques like CTP or DSC-MRI require the administration of contrast agent and additional tracer kinetic analysis [7,8].

Arterial Spin Labeling (ASL) represents a completely non-invasive MRI technique. By magnetically labeling the blood of brain feeding vessels it is possible to measure cerebral perfusion without external contrast agent injection [9]. Usually, blood is being labeled proximal to the imaging volume by inverting the blood water protons in the arteries of the neck. A certain amount of time is required for the labeled volume to flow into the brain. After this post-labeling delay images are acquired of the region of interest (label image). Another set of the same image volume is being acquired without prior labeling of the blood spins (control image). Subsequent subtraction of both label and control images eliminates static tissue signal and results in a perfusion weighted image. Different approaches exist that are capable of restricting the labeling to few or even single arteries [10]. A recently developed method called superselective ASL allows labeling of individual arteries, thus making it possible to visualize the perfusion territory of only a single vessel [11]. By changing the gradient moment of transversal gradients perpendicular to the flow direction, superselective ASL can adapt the labeling focus to the lumen of a selected artery ranging in size of the major brain feeding arteries to small branches distal to the circle of Willis. This makes it possible to label the blood in various vessels, e.g. intracranial-extracranial bypasses or AVMs.

This study investigates the feeding vasculature to intracranial meningiomas. Superselective ASL was used to identify and quantify the contribution of blood supply of individual vessel to intracranial meningiomas. The aim of this study was to demonstrate the feasibility of superselective ASL and quantification of vessel supply in intracranial meningiomas and to test its potential ability to serve as a screening tool prior to DSA.

## Material and methods

### Patients

The ethics committee of the Christian-Albrecht-University Kiel approved the study. All patients gave informed consent in oral and written form. We prospectively included consecutive patients presenting for surgical resection of intracranial meningiomas.

### Imaging

Superselective ASL [11] was used to visualize the contribution of blood supply to the tumors for both internal carotid arteries (ICA), both external carotid arteries (ECA) and the vertebral or the basilar artery (VA/BA), depending on individual anatomy using the following parameters: FOV 220 x 220 x 104 mm^3^, voxel size 2.7 x 2.7 x 6 mm^3^, gradient echo planar read-out, labelling duration 1.65 s, postlabelling delay 1.525 s with background suppression, 15 slices and 20 averages of label and control images. Scan time was approximately 2:40 minutes per vessel. Planning of the labeling spot was performed based on time-of-flight (TOF) MR angiography images and corresponding maximum intensity projections (MIP) placed over the neck of the subject. The scan parameters were as follows: field-of-view (FOV) 180 x 180 mm^2^, voxel size 0.9 x 1.2 x 3 mm^3^, 30 slices, 20° flip angle and pulse repetition time (TR)/echo time (TE) was 15/3.2 ms, resulting in approximately 1:30 min scan time. All measurements were performed on a clinical Philips 3 Tesla Achieva scanner (Philips, Best/The Netherlands).

### Image post processing

Perfusion images of individually labeled arteries were generated by pair-wise subtraction of label and control images and subsequent averaging. The perfusion territory images were then color-coded and combined into a single frame. For anatomical delineation a complete diagnostic set of images including T1w contrast enhanced images acquired prior to surgery during the clinical work-up was available for all patients. For further tumor characterization tumor volume was measured on appropriate T1w contrast enhanced images by delineating the tumor on every slice and multiplying the sum with slice thickness by one reader.

### Image analysis

Perfusion images were used for subsequent rating. Two readers (U.J.K. and M.H.) with 8 and 10 years of experience in neuroradiology and ASL imaging rated the extent of blood supply to every tumor and from each vessel as follows in a semiquantitative fashion: none, <50% or >50% of the tumor volume. These categories were chosen according to previous published values [12] because exclusive [3] or predominant [12] supply from the ECA is required for successful and safe embolization. A consensus session was held in which the differing results were discussed if disagreement was present between the raters until a consensus was reached. Interrater agreement was calculated using Cohen‘s κ [13]. Values of 0–0.20 were regarded as slight, 0.21–0.40 as fair, 0.41–0.60 as moderate, 0.61–0.80 as substantial, and 0.81–1 as (almost) perfect (R, version 3.5.0, R Foundation for Statistical Computing, Vienna, Austria).

### Validation

To validate superselective ASL we could use the respective results of two patients receiving additional gold standard diagnostic DSA. To this end the results of ASL and DSA were rated in the same manner described above by the two raters.

## Results

32 patients (29 females, 3 males, age 61 ± 13 years) were prospectively included. One patient was excluded due to histological diagnosis of metastatic disease. The remaining patients were harboring a total of 42 (median 1, range 1–7) intracranial tumors. 31 meningiomas could be histologically confirmed. In cases histology was not obtained (n = 11) image appearance of the tumors was consistent with meningioma (dural based well defined mass, isointense to gray matter on T1w and T2w images, intense homogenous enhancement, hyperostosis or calcifications on CT). Mean tumor volume was 43 ± 53 cm^3^ (range 0.35 to 231 cm^3^). For tumor location and size see **Table 1**.

**Table 1:** Individual vessel supply and size (mean ± SD) of the meningiomas.

Location	Total	Right ICA		Left ICA		Right ECA		Left ECA		AV		Volume (cm^3^)
		<50%	>50%	<50%	>50%	<50%	>50%	<50%	>50%	<50%	>50%	
Sphenoid wing	13	**4**	**6**	**4**	**1**	**4**	**2**	**0**	**4**	**0**	**0**	54 ± 41
Convexity	11	**1**	**1**	**1**	**1**	**0**	**6**	**1**	**3**	**0**	**0**	47 ± 56
Posterior fossa	6	**1**	**0**	**2**	**0**	**0**	**1**	**1**	**1**	**2**	**0**	31 ± 46
Tuberculum sellae	4	**0**	**3**	**1**	**1**	**0**	**0**	**1**	**0**	**0**	**0**	6 ± 4
Tentorium	3	0	1	1	1	2	1	0	0	1	0	19 ± 20
Falx	2	0	1	1	0	0	1	0	0	0	0	117 ± 162
Ventricle	1	0	1	0	0	0	0	0	0	0	0	95
Planum sphenoidale	1	0	1	1	0	0	0	1	0	0	0	54
Optic nerve	1	0	0	0	1	0	0	0	0	0	0	2
**Total**	**42**	**6**	**14**	**11**	**5**	**6**	**11**	**4**	**8**	**3**	**0**	**43 ± 53**

Note that some categories are not exclusive, some tumors are supplied by more than one vessel, thus the numbers may exceed the total number of meningiomas in the respective category.

**Fig 1:**
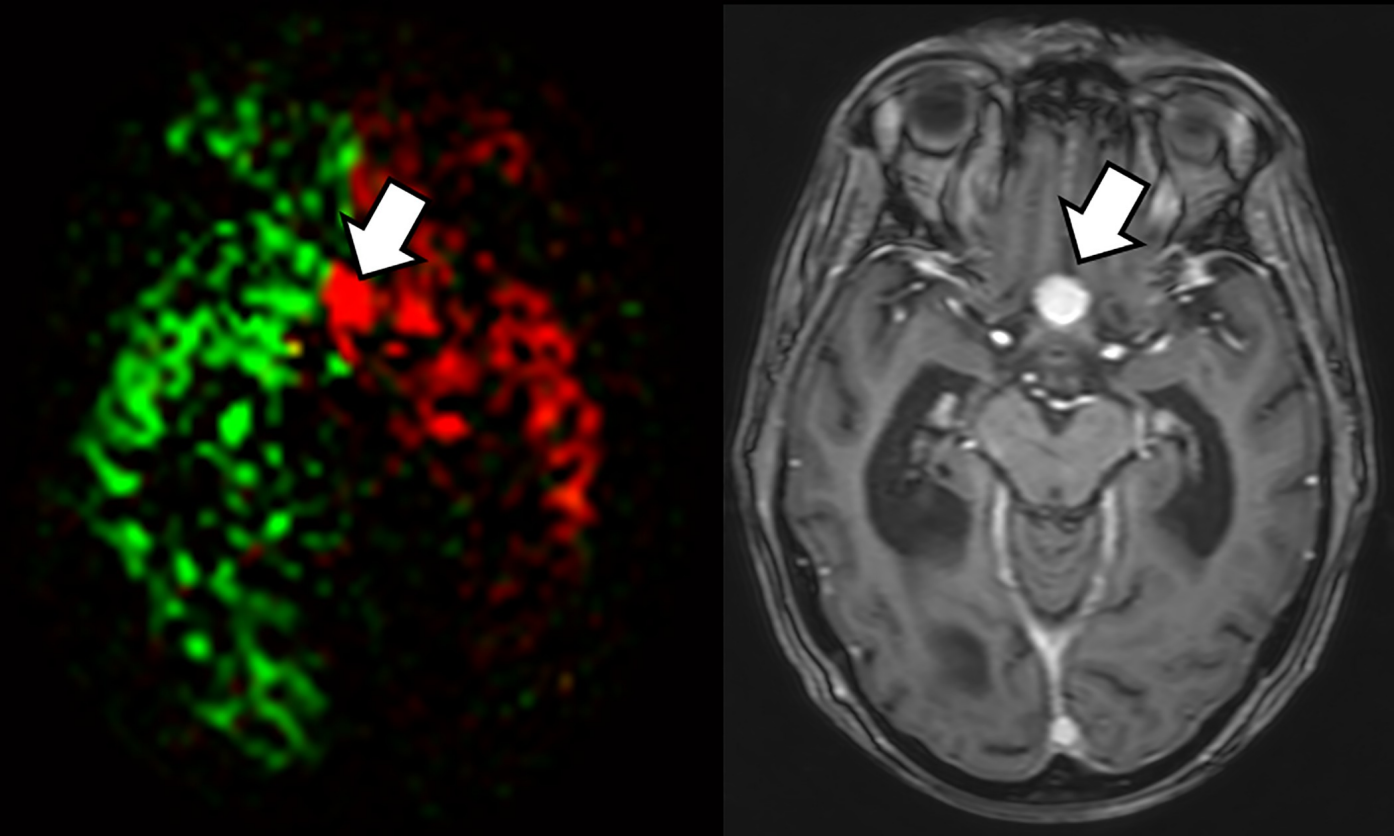
The smallest meningioma (arrow, 1.28 cm^3^) in which a perfusion signal could be detected was located at the tuberculum sellae (left: merged perfusion image, right: contrast enhanced T1w axial image). >50% contribution from the left internal carotid artery (red).

Superselective ASL was successfully performed in all patients, i.e. a perfusion signal could be detected in brain parenchyma supplied by the labelled vessels or in case of the ECA in extracranial scalp branches of the ECA. With the exception of three meningiomas (posterior fossa (n = 2), size 0.35 cm^3^ and 7.35 cm^3^, convexity (n = 1), size 1.34 cm^3^) a perfusion signal could be detected in all tumors. The smallest meningioma in which an ASL perfusion signal could be detected was 1.28 cm^3^ in size and located at the tuberculum sellae (**Fig 1**).

**Fig 2:**
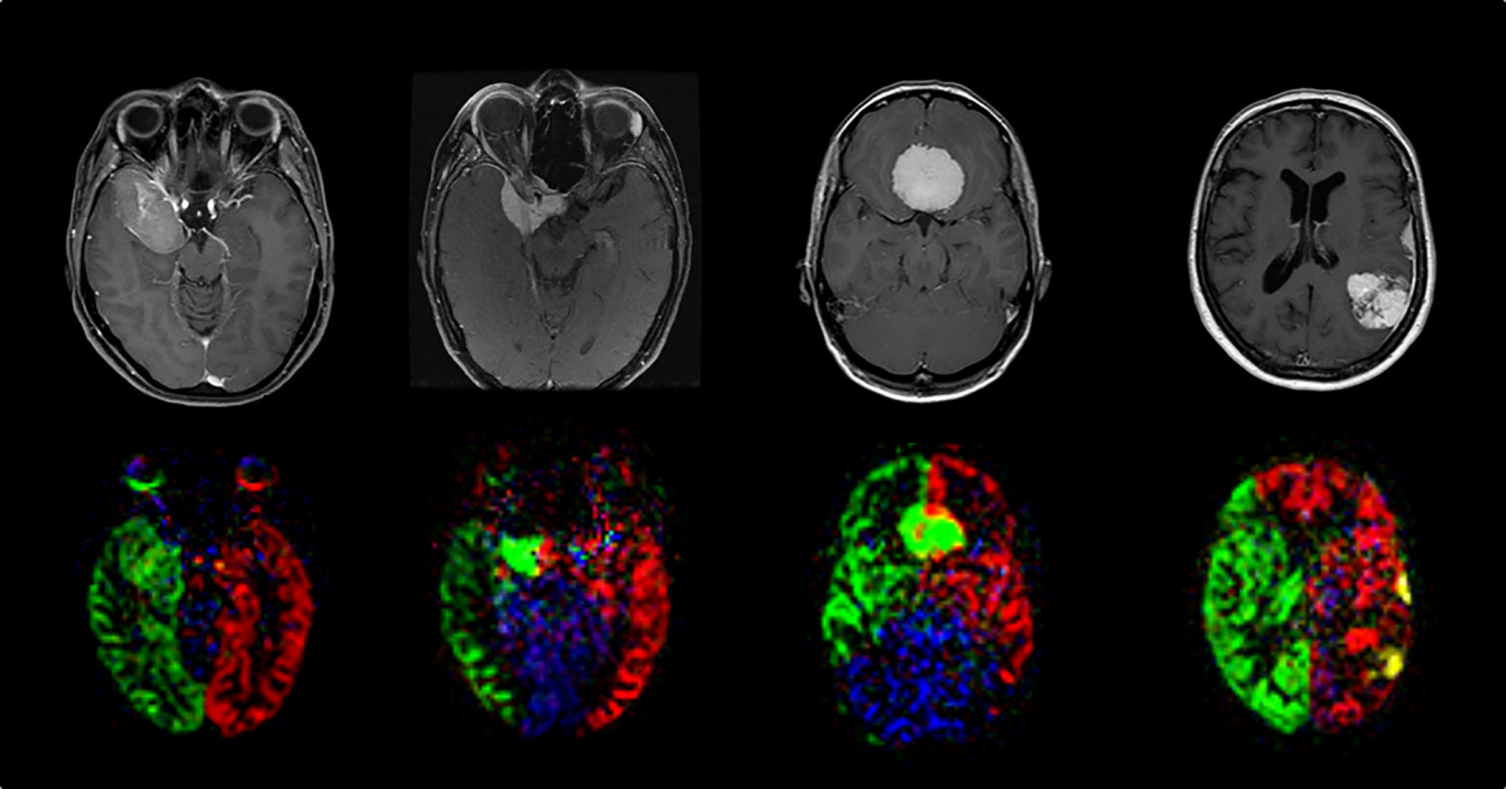
Example cases. Anatomical T1w images (first row) alongside the corresponding perfusion territories obtained by superselective ASL, color-coded and merged in a single frame (second row). **First column**: Exclusive (>50%) contribution of the right internal carotid artery (green), no contribution from other sources. **Second column**: >50% contribution from the right internal carotid artery (green), <50% contribution from the left internal carotid artery (red). **Third column**: >50% contribution from the right internal carotid artery (green), <50% contribution from the left internal carotid artery (red). **Fourth column**: Exclusive (>50%) contribution from the left external carotid artery (yellow) in the frontal convexity meningioma, >50% contribution from the left internal carotid artery (red), <50% contribution from the left external carotid artery (yellow). There was contribution from the vertebral arteries/basilar artery (blue) in these cases.

40% of the tumors (n = 17) had <50% supply from the ICA, 45% (n = 19) had >50% supply from the ICA. 17% (n = 7) had exclusive ipsilateral ICA supply. 24% of the tumors (n = 10) had <50% supply from the ECA, 45% (n = 19) had >50% supply from the ECA. 30% (n = 13) had exclusive ipsilateral ECA supply. 36% (n = 15) of the meningiomas had vessel supply from both ECA and ICA sources. 7% (n = 3) of the meningiomas had supply from the BA or VA but only one of them exclusively. 26% (n = 11) of the meningiomas had bilateral vessel supply. 50% (n = 21) of the meningiomas exhibited mixed vascular supply from multiple vessels. (**Fig 2**, **Tables 1 and 2**). Interrater agreement was κ = 0.73 or substantial.

**Table 2:** Pattern of vessel supply of the meningiomas.

Location	Total	Unilateral ECA supply	Unilateral ICA supply	ICA+ECA supply	Bilateral supply	BA/VA supply	Multiple vessel supply
Sphenoid wing	13	2	1	8	4	0	10
Convexity	11	9	0	1	2	0	2
Posterior fossa	6	1	1	2	0	2	2
Tuberculum sellae	4	0	3	1	1	0	2
Tentorium	3	0	0	2	2	1	3
Falx	2	1	0	0	1	0	1
Ventricle	1	0	1	0	0	0	0
Planum sphenoidale	1	0	0	1	1	0	1
Optic nerve	1	0	1	0	0	0	0
**Total**	**42**	**13**	**7**	**15**	**11**	**3**	**21**

Note that some categories are not exclusive, e.g. multiple vessel supply may include tumors with bilateral supply, thus the numbers may exceed the total number of meningiomas in the respective category.

**Fig 3:**
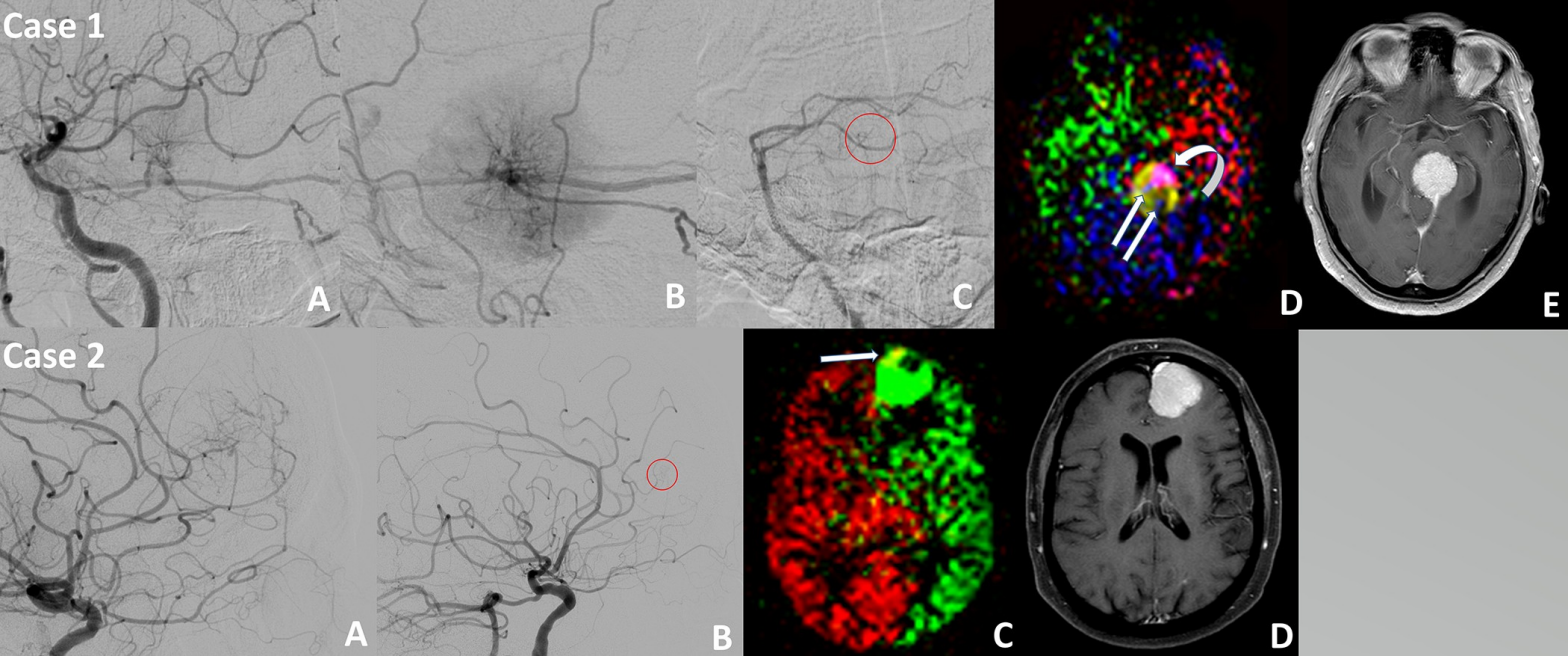
Correlation of DSA with superselective ASL. **Case 1**: Lateral projection of diagnostic DSA with <50% contribution of the left internal carotid artery (**A**), >50% contribution of the left external carotid artery (**B**) and only little (<50%) contribution (circle) from branches of the posterior cerebral artery (**C**). The tumor perfusion is visible as a tumor blush, most prominently visible in **B**. Corresponding color encoded perfusion maps merged in a single frame (**D**). Note matching extent of arterial supply on DSA and ASL (left internal carotid artery <50% (light red, curved arrow), left external carotid artery >50% (yellow) and vertebral artery <50% (blue, double arrow)). Corresponding contrast enhanced T1w axial image of the left tentorial meningioma (**E**). **Case 2:** Lateral projection of diagnostic DSA with >50% contribution of the left internal carotid artery (**A**) and only little contribution (circle) of the left external carotid artery (**B**). Corresponding color encoded perfusion maps merged in a single frame (**C**). Note matching extent of arterial supply on DSA and ASL (left internal carotid artery >50% (red), right internal carotid artery <50% (yellow, arrow) and no contribution from other sources). Corresponding contrast enhanced T1w axial image of the left frontal convexity meningioma (**D**).

Correlation of superselective ASL with DSA demonstrated complete agreement with our predefined categories (interrater agreement κ = 1 and κ = 0.81) and both cases are depicted in **Fig 3**.

## Discussion

We could demonstrate the feasibility of superselective ASL in patients with intracranial meningiomas to identify the feeding vasculature. Although one previous study already used this approach [6] our study was the first to incorporate a semiquantitative approach to quantify the extent of supply of different feeding vessels.

Previous ASL-work has been dedicated to the topic of vascular supply to meningiomas: Selective ASL with labelling of the ECA was applied by Sasao and coworkers [14]. In conjunction with DSA they were able to depict individual perfusion territories of the ECA. The methodology used in the study restricted the use of ASL to the flow territories of the ECA. Lum and coworkers used DSC-MRI with intraarterial bolus injection to differentiate between pial and dural supply [8]. Their approach is invasive, requires contrast injection and further postprocessing. However, they were able to convincingly distinguish between supply from dural and pial sources within the meningiomas. Lu et al. could demonstrate the feasibility of selective ASL in intracranial meningiomas [6]. They used TOF angiography as the validation method. Our study corroborates their approach even in small meningiomas. However, TOF angiography does not allow for quantification of the supply of the individual vessels. ASL has also been used to perform meningioma grading and identify high grade aggressive forms [15]. Non selective ASL was used by Yoo et al. to assess meningioma vascularity in comparison to DSA and found a high positive correlation between the two modalities [16]. Kikichi et al. investigated the added values of ASL as an additional tool to detect recurrent meningioma and found an increased detection rate among non-specialists [17].

Presurgical meningioma embolization can be performed in intracranial meningiomas to prevent or reduce hemorrhagic complications or as a palliative therapy if the patient is unfit for surgery [18]. However, presurgical meningioma embolization is discussed controversially [19, 20]. No consensus on the benefit of this invasive procedure has been reached since the reported blood loss is variable in the literature and if present border line significant [21, 22]. However, a certain consensus has been reached on which patients are amenable to presurgical meningioma embolization: Patients eligible for embolization will have an exclusive [3] or predominant [12] external carotid artery supply.

Additionally, ASL could be helpful when doubts on meningioma attachment (e.g. frontal convexity or falcine attachment) exist or the meningioma has potentially recruited new, atypical feeders. The results of ASL could then be used to plan the surgical approach.

Our study has some limitations. In recent years the number of requests for presurgical meningioma embolization or DSA has dramatically dropped in our institution, too. Validation with DSA could only be performed in two patients for the above listed reasons. However, we observed perfect correlation between DSA and ASL with our predefined categories. However, due to the limited number of cases this has to be regarded with caution. Furthermore, previous confirmatory work could already demonstrate agreement between DSA and ASL in meningiomas [14] and feedings vessel contribution in cerebral arterio-venous malformations [23]. Because DSA and presurgical meningioma embolization is restricted to selected cases ASL could serve as a screening method to identify patients most likely to benefit from DSA and subsequent embolization.

Three meningiomas did not display a perfusion signal. These three tumors were small, however, even smaller meningiomas displayed a perfusion signal. Potentially, factors such as calcification or other tumor properties of unknown relevance in this context also play an important role. We render it unlikely that technical settings like postlabelling delay may have a large effect. In the set-up for the study the postlabelling delay was adjusted to find an optimum and if tumor perfusion was initially unsuccessful during the study the postlabelling delay were adjusted with non to very modest effect (data not shown). The absence of such an effect was also observed by others [23].

ASL perfusion imaging suffers from a low signal-to-noise ratio (SNR) intrinsic to this method. The blood is labeled outside the imaging region and flows into the region of interest while the blood spins already starts to relax. Moreover, ASL relies on the subtraction of labeled and non-labeled images. As a consequence, rather large voxels are acquired to ensure sufficient SNR in clinically acceptable scan times. Very recently, Kim et. al [24] demonstrated the possibility of training a convolutional neural network to generate ASL perfusion images with higher accuracy and robustness by using a smaller number of subtraction images. This may also offer the possibility to use a similar network for the creation of perfusion images with higher spatial resolution. However, the generation of training data will still require longer scan times for volunteers and patients as this dataset has to be independent of the primary dataset acquired here.

Further, blood supply of cranial nerves by ECA branches [25] are not depictable by the technique used here but is necessary to prevent non target embolization. This information can be reliably obtained with DSA. This makes DSA irreplaceable in the presurgical work-up of meningiomas scheduled for embolization. However, superselective angiography [26] could potentially substitute DSA if further confirmatory work is done. Due to the recruitment process only patients presenting for surgery were included. Meningiomas in unusual localization are underrepresented and histological confirmation is only available for resected, i.e. mostly large or symptomatic meningiomas.

## Conclusion

Superselective ASL allows semiquantitative depiction of blood supply to intracranial meningiomas. It may serve as a screening method for patients who may benefit from DSA and embolization. It may also be of special interest in future MR guided invasive embolization procedures [27]. More validation against DSA in larger cohorts of patients is necessary.
